# COMMUNITY CAPACITY AND SOCIAL PARTICIPATION AMONG COMMUNITY-DWELLING MIDDLE-AGED AND OLDER CHINESE

**DOI:** 10.1093/geroni/igz038.3057

**Published:** 2019-11-08

**Authors:** Min Li, Fang-Yi Huang

**Affiliations:** 1 University of Florida, Gainesville, Florida, United States; 2 Trinity Washington University, College Park, Maryland, United States

## Abstract

Objectives. This study investigates how social participation of the aging population is associated with the community capacity, measured by the number of amenities and organizations within the community. Method. Using nationally representative survey data from the China Health and Retirement Longitudinal Study (2011), this study examines the availability of community amenities and organizations in rural and urban areas, and investigates the associations between community capacity and social participation among the middle aged and older Chinese using multilevel analysis. Results. The results of this study indicate that both community amenities and community organizations are positively associated with the social participation of the middle-aged and older Chinese. Additionally, the association between community organizations and the frequency of formal social participation is stronger among urban communities than rural ones, even after controlling for the individual-level socioeconomic status and health conditions. Conclusion. This study highlights the importance of building the community capacity by developing community-based grassroots organizations to promote the social engagement and participation of the aging population.

